# Effects of age on electrophysiological correlates of speech processing in a dynamic “cocktail-party” situation

**DOI:** 10.3389/fnins.2015.00341

**Published:** 2015-09-29

**Authors:** Stephan Getzmann, Christina Hanenberg, Jörg Lewald, Michael Falkenstein, Edmund Wascher

**Affiliations:** Aging Research Group, Leibniz Research Centre for Working Environment and Human FactorsDortmund, Germany

**Keywords:** speech perception, attention, cocktail party, aging, event-related potentials

## Abstract

Successful speech perception in multi-speaker environments depends on auditory scene analysis, comprising auditory object segregation and grouping, and on focusing attention toward the speaker of interest. Changes in speaker settings (e.g., in speaker position) require object re-selection and attention re-focusing. Here, we tested the processing of changes in a realistic multi-speaker scenario in younger and older adults, employing a speech-perception task, and event-related potential (ERP) measures. Sequences of short words (combinations of company names and values) were simultaneously presented via four loudspeakers at different locations, and the participants responded to the value of a target company. Voice and position of the speaker of the target information were kept constant for a variable number of trials and then changed. Relative to the pre-change level, changes caused higher error rates, and more so in older than younger adults. The ERP analysis revealed stronger fronto-central N2 and N400 components in younger adults, suggesting a more effective inhibition of concurrent speech stimuli and enhanced language processing. The difference ERPs (post-change minus pre-change) indicated a change-related N400 and late positive complex (LPC) over parietal areas in both groups. Only the older adults showed an additional frontal LPC, suggesting increased allocation of attentional resources after changes in speaker settings. In sum, changes in speaker settings are critical events for speech perception in multi-speaker environments. Especially older persons show deficits that could be based on less flexible inhibitory control and increased distraction.

## Introduction

The ability to listen to one talking person while other people are talking or laughing at the same time is “probably the best-known real life example of selective attention” (Pashler, 1998, p. 37) and has been termed “cocktail-party” effect (Cherry, 1953; for review, see Bronkhorst, 2000). Successful speech perception in multi-speaker environments requires (a) auditory scene analysis, including auditory stream segregation, and grouping (Bregman, 1994) and (b) focusing auditory attention on the speaker of interest while simultaneously suppressing concurrent sound sources (for review, see Shinn-Cunningham, 2008). It is assumed that auditory object formation and selective attention are closely related (Ihlefeld and Shinn-Cunningham, 2008; Shinn-Cunningham and Best, 2008). Moreover, the two processes are not automatic or invariant, but (at least partly) attention-based and time-consuming (Cusack et al., 2004; Shinn-Cunningham and Best, 2008; for electrophysiological evidence, see Kerlin et al., 2010; for review, see Fritz et al., 2007). Accordingly, continuity in auditory scenery has been found to result in increasing efficacy in object selection and in continuous focusing of attention toward the features of a relevant source (e.g., a speaker's position or voice). In contrast, changes in auditory scenery require renewed object formation and attentional re-focusing (Best et al., 2008).

In addition to changes triggered by the auditory environment, a deliberate switch from one speaker of interest to another, previously unattended speaker also requires attentional re-focusing. Employing a dichotic-listening paradigm with two spatially separated speakers, Koch et al. (2011) examined the mechanisms of intentional switching of selective auditory attention. The authors found substantial switch costs in trials after a change, indicated by higher error rates and reaction times relative to the pre-change level. A temporal preparation of the attentional re-focusing (by providing cues indicating changes) reduced these switch costs only in part, suggesting a form of sluggishness (“inertia”) in the cognitive control of selective attention (Koch et al., 2011; regarding re-focusing of auditory spatial attention, see also Mondor and Zatorre, 1995; Kidd et al., 2005; Singh et al., 2008). In addition, switch costs are assumed to result from a persisting activation of a previously attended sound source as well as from a persisting inhibition of a previously unattended sound source (for review, see Koch et al., 2010). Finally, costs of reconstruction of speech information (that are missed due to the change of attention) may play a role (Shinn-Cunningham and Best, 2008). Thus, a recent study in which listening costs associated with shifts in spatial attention were tested in a dynamic multi-speaker environment emphasized the role of working memory in maintaining goal-relevant information and meaning extraction (Lin and Carlile, 2015). In sum, changes in dynamic auditory sceneries and switches of auditory attention appear to be critical events for speech perception in the presence of concurrent speech information.

This may be true especially in aging. Older adults typically show reduced speech-in-noise perception abilities (Burke and Shafto, 2008), and often report difficulties in understanding what has been said in cocktail-party situations (for review, see Wingfield and Stine-Morrow, 2000). These deficits are mainly based on age-related changes in peripheral hearing (presbycusis) and in central auditory processing (Humes and Dubno, 2010). In addition, declines in general cognitive abilities such as working memory capacity, inhibitory control, and information processing speed (Van der Linden et al., 1999) are assumed to contribute to deficits to manage speech perception in multi-speaker environments (for review, see Schneider et al., 2010). In particular, reduced inhibitory control might lead to attentional deficits, according to the inhibition deficit hypothesis (Hasher and Zacks, 1988; Hasher et al., 2007). In line with this hypothesis, we observed age-related differences in event-related potentials (ERPs) in two recent studies, in which we investigated speech perception in a simulated multi-speaker scenario. In these studies, older adults showed reduced attentional and inhibitory control and reduced speech processing abilities relative to younger adults, as indicated by a less pronounced N2 and N400 complex (Getzmann et al., 2014a). Moreover, a comparison of older high-performing and low-performing adults revealed a pronounced frontal P2 component in the high-performing group (Getzmann et al., 2015a). In line with the decline-compensation hypothesis (Wingfield and Grossman, 2006), this was interpreted as an increased allocation of mental resources for compensation of deficiencies in peripheral and central auditory processing. Together with previous work (for review, see Schneider et al., 2010), these findings showed that focusing attention on a speaker of interest (while suppressing irrelevant auditory information in multi-speaker environments) could be more challenging for older than younger adults.

Assuming that speech-perception in a more dynamic (i.e., highly variable) multi-speaker environment requires extra cognitive resources for auditory object formation and selective attention (e.g., Lin and Carlile, 2015), it could be expected that older adults have even more difficulties with changes in speaker settings than younger ones. In line with this assumption, the results of a recent study indicated that rare and irregular shifts in target-speaker position decrease performance of older adults more than that of younger adults, probably due to a delay in the attention switching mechanism (Getzmann et al., 2015b). Thus, reduced attentional flexibility in combination with reduced inhibitory control could result in deficits in re-orientation (Salthouse, 1996; Wecker et al., 2005; for review, see Kok, 2000) that may lead to a potential loss of speech information after a change in speaker setting. Accordingly, segregation of speech signals of interest from concurrent speech in the presence of a speech masker appeared to be more “sluggish” in older than in younger adults (Schneider et al., 2010; Ezzatian et al., 2015).

In the present study, we addressed this issue and tested the ability of younger and older adults to switch between various speakers in a multi-speaker environment. The focus was on the effect of changes in target speaker settings on speech perception. For this purpose, we analyzed behavioral and electrophysiological measures obtained prior to and after a change. As in our previous studies, a simulated stock-market situation was employed in which sequences of short company names and values were simultaneously presented via four different loudspeakers at different locations (Getzmann et al., 2014a, 2015a). The participants attended to the name of a target company and judged whether the value of the target company was above or below a given level. While in the previous studies the target speaker changed in every trial, here we established a situation in which the target speaker setting was kept constant for a while and then changed. Thus, the target company was spoken by the same speaker and at the same position for a variable number of trials, and then the target-speaker voice or position, or both, changed. These changes required (a) the re-focusing of attention to the new, previously irrelevant, speaker of interest and (b) the inhibition of concurrent speakers that might have been relevant in pre-change trials. By comparing the error rates in trials before and after a change, we determined the switch costs in younger and older participants. We assumed that there is a decrease in performance in post-change trials relative to the pre-change level and that this decrease is stronger in the older, than in the younger, group.

The electrophysiological correlates of change processing were investigated by analysis of ERPs. The most prominent ERPs associated with early stimulus processing are the P1, reflecting rather exogenous influences (Grunwald et al., 2003), and the N1, reflecting the earliest stages of attentional focusing and orientation to novel stimuli (Hillyard et al., 1973; Näätänen and Picton, 1987). Correlates of subsequent processing stages are represented by the P2, N2, and N400 deflections that depend more on the listener's attentional state: The P2 is associated with target detection and attentional allocation (Potts, 2004), while the N2 usually reflects inhibitory control and suppressing irrelevant information (Folstein and Van Petten, 2008). Correlates of language perception and extraction of semantic speech information are given by the N400 (e.g., Kutas and Federmeier, 2011; Davis et al., 2013). Finally, the late positive component (LPC) was analyzed, which has been associated with context updating and evaluation and processing of stimulus meaning (Juottonen et al., 1996). This component has recently been found to be sensitive to age in speech perception (Davis and Jerger, 2014).

## Materials and methods

### Subjects

A total of 22 younger (11 female, mean age 24.0 years, age range 18–35 years) and 22 older (11 female, mean age 64.3 years, age range 55–72 years) adults participated in the study. The younger subjects were students at local universities; the older subjects were recruited by advertisements in regional daily newspapers. All participants reported to be healthy, free of medication during the experimental sessions, and without any history of neurological, psychiatric, or chronic somatic problems. All participants wrote with their right hand. Subjects underwent a pure-tone audiometry (Oscilla USB 330; Inmedico, Lystrup, Denmark) at 125–8000 Hz. Audiograms indicated mild to moderate presbyacusis in the older group. However, in all participants hearing was normal (thresholds ≤ 30 dB hearing level) in the range below 4000 Hz. Prior to experimentation, all subjects gave their written informed consent to participate in the study. The study conformed to the Code of Ethics of the World Medical Association (Declaration of Helsinki) and was approved by the local Ethical Committee of the Leibniz Research Centre for Working Environment and Human Factors, Dortmund, Germany.

### Apparatus, stimuli, and task

**Figure 1:**
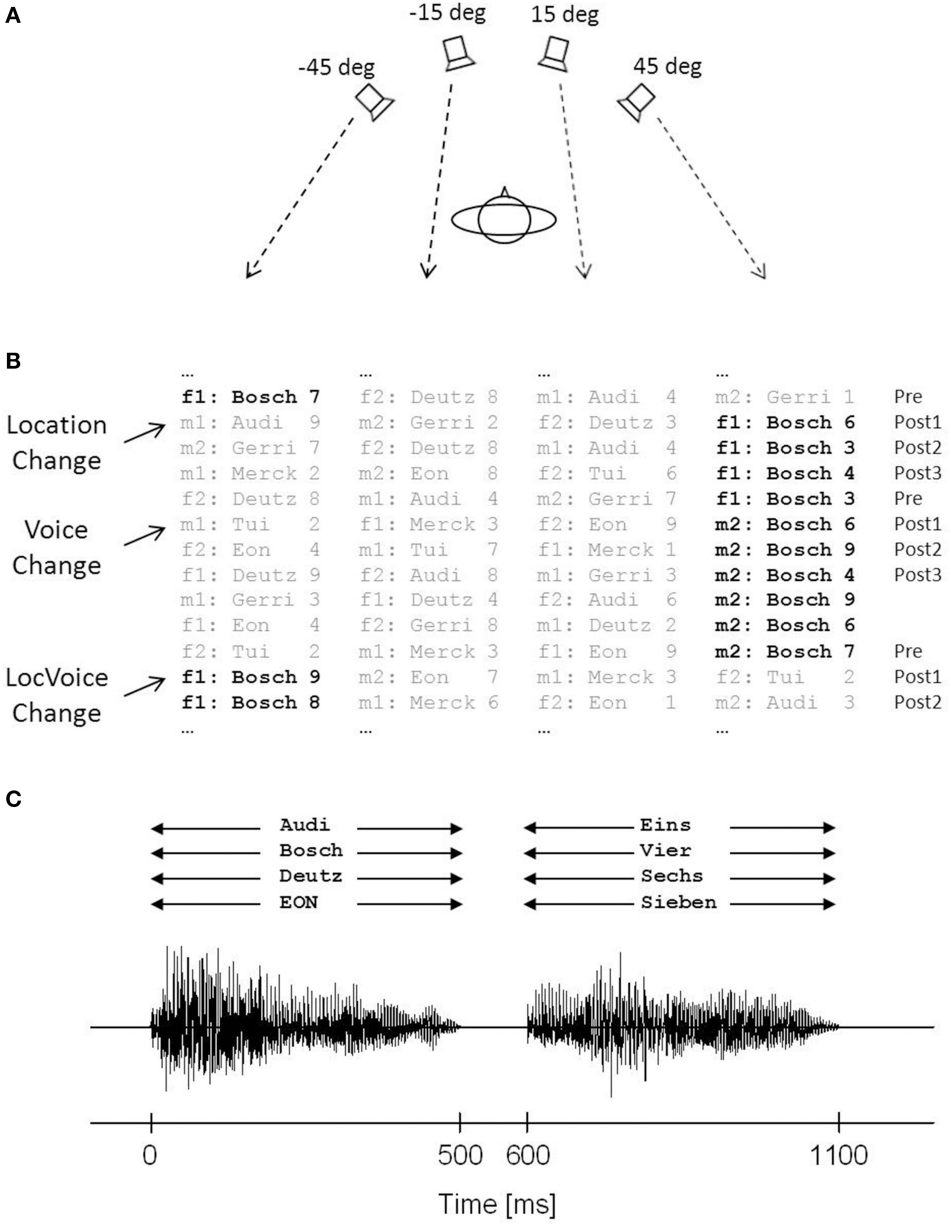
**Schematic illustration of the simulated stock-price monitoring scenario. (A)** Four loudspeakers were mounted at different locations to the left and right of the subject's median plane. Stimuli were displayed simultaneously via all loudspeakers. **(B)** Stimuli were spoken by two female (f1, f2) and two male (m1, m2) speakers and consisted of sequences of short company names and numbers. The participants responded to the value of a given target company (here “Bosch,” in bold print), while all other company names had to be ignored. The location of the target company (Location Change), the speaker voice (Voice Change), or both (LocVoice Change) changed following a pseudo-random scheme. Analyses were focused on sequences preceding (Pre) and following a change trial (Post1, Post2, Post3). **(C)** Superimposed acoustic waveforms of the four streams of words (here: company names “Bosch,” “Deutz,” “EON,” and “Audi,” and numbers “eins,” “vier,” “sechs,” and “sieben”) presented at different locations.

Experiments were conducted in a dimly illuminated, video-controlled, electrically shielded, and sound-proof room (5.0 × 3.3 × 2.4 m^3^) with pyramid-shaped foam acoustic panel on ceiling and walls. The floor was lined with a sound-absorbing woolen carpet. The ambient background noise level was below 20 dB(A). The subject was seated in a comfortable, vertically adjustable chair in the center of the room. The position of the head was held constant by a chin rest. To create a free-field scenario, stimuli were presented via four broad-band loudspeakers (SC 5.9, Visaton, Haan, Germany), mounted in front of the subject at a distance of 1.5 m from the center of the head (Figure 1A). The loudspeakers were arranged at ear level in the horizontal plane and were located at −45°, −15° (left), 15°, and 45° (right) azimuth. The loudspeakers were selected on the basis of similar efficacy and frequency response curves to minimize output and fidelity differences. Loudspeakers were controlled by custom-made amplifiers and software. Speech stimuli were digitally recorded at 48 kHz sampling rate and 16-bit resolution in a sound-proof and anechoic environment using a freestanding microphone (MCE 91, Beyerdynamic, Heilbronn, Germany), a mixing console (1202-VLZ PRO, Mackie, Woodinville, WA), and an external soundcard (Terrasoniq TS88 PCI, TerraTec Electronic, Nettetal, Germany). Stimuli were processed offline using CoolEdit 2000 software (Syntrillium Software Co., Phoenix, AZ, USA) and converted to analog form via a computer-controlled external soundcard (Terrasoniq TS88 PCI, TerraTec Electronic, Nettetal, Germany).

The speech stimuli consisted of eight one or two syllable names of companies (Audi; Bosch; Deutz; Eon; Gerri; Otto; Merck; Tui) and eight one or two syllable German numerals (Eins [1]; Zwei [2]; Drei [3]; Vier [4]; Sechs [6]; Sieben [7]; Acht [8]; Neun [9]). These stimuli were spoken by two male and two female monolingual native German adults of young and middle age, without any dialect or speech disorders. The fundamental frequencies of their voices were 123 and 126 Hz for the male speakers, and 162 and 171 Hz for the female speakers. The overall duration of each company name and each number was 500 ms.

Word pairs of a company's name and a numeral simulating its stock price (e.g., “Bosch—eins” [“Bosch—one”] or “Deutz—acht” [“Deutz—eight”]) were presented to the participants. Company names and numerals were separated by a 100-ms silent interval, such that the overall duration of each word pair was 1100 ms. The word stimuli were presented at a level of 65 dB(A). Each company name was combined with each of the eight numerals, thus resulting in 64 word pairs. Participants were instructed to attend to a given target company (either “Bosch” or “Deutz,” balanced across participants). Using a two-alternative forced-choice paradigm, they had to press the upper button of a keypad when the value of the target company was above five (values “6,” “7,” “8,” or “9”; each in 12.5% of trials), and the lower button when the value was below five (values “1,” “2,” “3,” or “4”; each in 12.5% of trials), using the index and the middle finger of the dominant hand. The participants were instructed to respond after presentation of the company value, but before the onset of the next trial. Each combination of target company and numeral was spoken by the same speaker and was presented via the same loudspeaker for a variable number of trials. Concurrent company names paired with different numerals were simultaneously presented via the three other loudspeakers (Figure 1B). The onsets and offsets of the mixture of the four company names and numbers were approximately aligned in time (Figure 1C). The subjects were instructed to ignore this concurrent speech information. To generate a dynamic multi-speaker scenario, speakers and locations of the concurrent companies and numerals randomly changed between trials in a way that within each trial (a) all four speakers were active, (b) each company name and value occurred only once, and (c) always two numerals were above and below the critical value of five. After three, four, five, or six subsequent trials (4.5 trials on average) the speaker or the location of the target company, or both speaker and location changed following a pseudo-randomized scheme.

In each session, a total of 1472 trials was presented in four blocks, each lasting about 20 min. The four blocks were separated by short rest breaks. Each trial lasted for 3 s, leaving 1.9 s after the end of the numeral for response. Among the 1472 trials, there were 324 change trials, in which either the speaker voice (108 trials), the speaker location (108 trials), or both voice and location (108 trials) changed. Among the 324 change trials, there were 81 changes in which the Post3 trial coincided with the Pre trial of the subsequent change. These Pre trials were not included in the analysis. No feedback was given at any time during the experiment. Prior to experimentation, the participants were familiarized with the task in a practice block of about 20 trials, in which sequences of target companies and numbers were presented isolated, without concurrent stimuli. The timing of the stimuli and the recording of the participants' responses were controlled by custom-written software.

### Data recording and analysis

#### Behavioral data

The present study was focused on age-related differences in the processing of changes in dynamic multi-speaker scenarios. Therefore, the three change conditions (voice, location, both) were pooled to improve the signal-to-noise ratio of the EEG data and to reduce the complexity of the analysis. The rates of correct responses of younger and older subjects were analyzed for trials preceding a change (Pre) and following a change (Post1, Post2, Post3). The rates of correct responses were subjected to a Two-Way repeated-measures analysis of variance (ANOVA) with the between-subjects factor Age (younger, older) and the within-subjects factor Sequence (Pre, Post1, Post2, Post3). In addition, possible effects of changes in speaker setting were assessed by subjecting the differences in correct responses (post-change minus pre-change) to a Two-Way ANOVA with the between-subjects factor Age (younger, older) and the within-subjects factor Sequence (Post1, Post2, Post3). Given that the participants were instructed to wait with their response until the company value was presented, we refrained from analysing response times. For comparison between sequence conditions, Bonferroni-corrected *Post-hoc t*-tests were applied, and only the corrected *p*-values are reported. Levene's tests were used to assess the homogeneity of variance, and the degrees of freedom were adjusted if variances were unequal. Only significant differences in homogeneity of variance between the groups were reported. Effect sizes were computed to provide a more accurate interpretation of the practical significance of the findings, using the partial eta-squared coefficient ($\eta_{p}^{2}$).

#### EEG data

The continuous EEG was sampled at 2048 Hz using 64 electrodes and a BioSemi amplifier (Active Two; Biosemi, Amsterdam, Netherlands). Electrode positions were based on the International 10–10 system. The amplifier bandpass was 0.01–140 Hz. Horizontal and vertical eye positions were recorded by electro-oculography (EOG) using 6 electrodes positioned around both eyes. Two additional electrodes were placed on the left and right mastoids. Electrode impedance was kept below 10 kΩ. The raw data were downscaled offline to a sampling rate of 1000 Hz, digitally band-pass filtered (cut-off frequencies 0.5 and 25 Hz; slopes 48 dB/octave), and re-referenced to the linked mastoid electrodes. The data were segmented into 2900-ms stimulus-locked epochs covering the period from −100 to 2800 ms relative to speech onset. Data were then corrected for ocular artifacts using the Gratton, Coles, and Donchin procedure (Gratton et al., 1983). Individual epochs exceeding a maximum-minimum difference of 200 μV and a maximum voltage step of 50 μV per sampling point were excluded from further analysis (automatic artifact rejection as implemented in the BrainVision Analyzer software, Version 1.05; Brain Products, Gilching, Germany). The remaining epochs were baseline corrected to a 100-ms pre-stimulus window relative to the onset of the speech stimulus. Trials containing correct responses were averaged for each participant.

Peaks of the different ERP deflections were defined as maximum positivity or negativity within particular latency windows of the specific waveforms with reference to the onset of the company name (P1: 10–110 ms at FCz; N1: 60–160 ms at Cz; P2: 145–245 ms at FCz; N2: 245–345 ms at FCz; N400: 380–480 ms at FCz). In addition, ERP deflections to the onset of the numeral were defined (P1n: 20–120 ms at FCz; N1n: 80–180 ms at Cz; time ranges relative to the numeral onset). ERP peak latencies were measured at electrode positions chosen to be commensurate with previous knowledge of the topographical scalp distribution of specific ERPs (Smith et al., 1980; Barrett et al., 1987; Näätänen and Picton, 1987; Lovrich et al., 1988; Friedman et al., 1993). The choice of these positions was confirmed by visual inspection of the grand average waveforms. Topographies of all seven ERPs were plotted for both age-groups and for Pre, Post1, Post2, and Post3 trials to underline possible differences in activation. In addition, difference waveforms (Post-change minus Pre-change) were computed for each participant and for Post1, Post2, and Post3 trials. The amplitude of the change-related ERPs (N400_diff_, frontal and parietal LPC_diff_; see below) were determined for each participant as the mean value of a 100-ms period centered at the peak latencies (N400_diff_: 390 ms at Pz; frontal LPC_diff_: 550 ms at AFz; parietal LPC_diff_: 740 ms at Pz). These latencies were determined as mean values averaged across all participants.

The ERP latencies were subjected to ANOVAs with the between-subjects factor Age (young, old) and the within-subjects factor Sequence (Pre, Post1, Post2, Post3). In order to test potential differences in topography, the ERP amplitudes were analyzed within arrays of 3 × 3 electrodes around the electrode position of maximal activation. These nine electrodes were grouped in form of a rectangular grid that consisted of three adjacent electrode positions in the frontal dimension and three adjacent electrode positions in the horizontal dimension. Electrode arrays around FCz (F3, FC3, C3, Fz, FCz, Cz, F4, FC4, C4) were used for P1, P2, N400, and P1n, arrays around Cz (FC3, C3, CP3, FCz, Cz, CPz, FC4, C4, CP4) for N2 and N1n, and an array around CPz (C3, CP3, P3, Cz, CPz, Pz, C4, CP4, P4) for N1. In addition, arrays around Pz (CP3, P3, PO3, CPz, Pz, POz, CP4, P4, PO4) were used for N400_diff_ and parietal LPC_diff_, and an array around FCz (AF3, FC3, CP3, AFz, FCz, CPz, AF4, FC4, CP4) for the frontal LPC_diff_. This resulted in two additional within-subjects factors, Frontality and Laterality, with Frontality being composed of frontal (e.g., F3, Fz, F4), fronto-central (FC3, FCz, FC4), and central (C3, Cz, C4) positions, and Laterality being composed of left (F3, FC3, C3), middle (Fz, FCz, Cz), and right (F4, FC4, C4) positions. The amplitude values of the ERPs were subjected to Four-Way ANOVAs (Age, Sequence, Frontality, and Laterality).

## Results

### Performance

**Figure 2:**
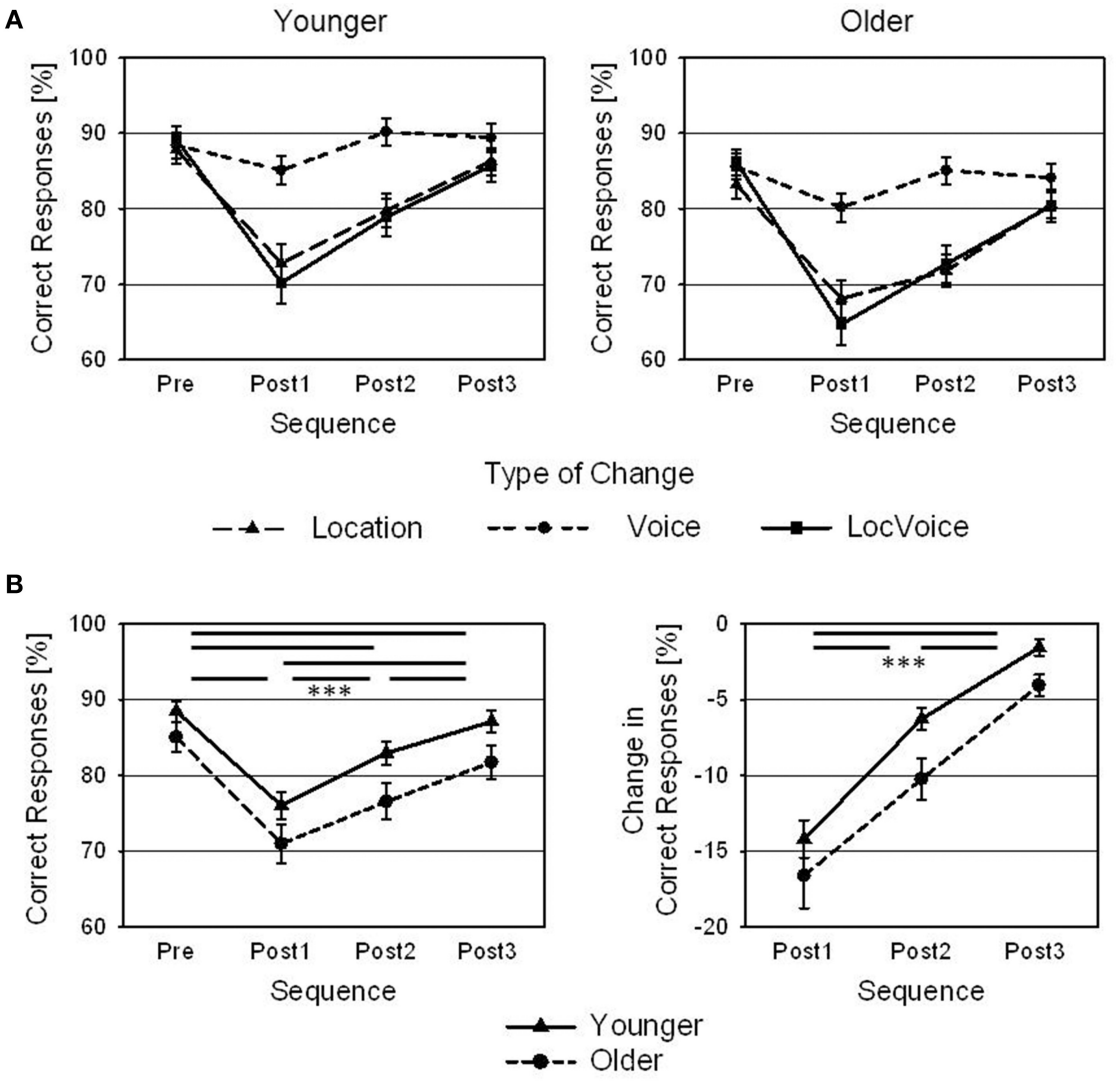
**Behavioral results. (A)** Rates of correct responses of younger and older adults for Pre, Post1, Post2, and Post3 sequences, and for changes in target speaker location, voice, and combined changes in location and voice (LocVoice). **(B)** Rates of correct responses (left panel) and changes in the rate of correct responses (same data normalized with reference to pre-change levels; right panel) of younger and older adults, averaged across all types of changes, shown for Pre, Post1, Post2, and Post3 sequences. Error bars are standard errors across participants (*N* = 22; ^***^p < 0.001).

Changes in target speaker location, target speaker voice, or both decreased the rates of correct responses of younger and older subjects, relative to the pre-change level. Although the amount of this decrease in performance differed between the different types of changes (withmost pronounced effects after changes in location and changes in both location and voice), the overall pattern was the same, namely a decrease in Post1 trials and a re-approach to the pre-change level thereafter (Figure 2A). Aggregated across the different types of changes, it lasted up to the Post3 trial until the rate of correct responses had re-approached the pre-change level (Figure 2B; left panel). A 4 × 2 ANOVA with Sequence (Pre, Post1, Post2, Post3) as within-subjects factor and Age as between-subjects factor indicated a main effect of Sequence [*F*_(3, 126)_ = 120.8; *p* < 0.001; $\eta_{p}^{2}=0.74$]. *Post-hoc* Bonferroni-corrected *t*-tests revealed significant differences in correct responses between all four sequences (Pre: 86.8%; Post1: 73.5%; Post2: 79.7%; Post3: 84.4%; all *p* < 0.001). Older participants performed slightly worse than the younger ones (78.6 vs. 83.6% correct responses), but the main effect of Age failed to reach clear statistical significance [*F*_(1, 42)_ = 3.8; *p* = 0.057; $\eta_{p}^{2}=0.08$]. There was no interaction of Age and Sequence (*p* > 0.05). Also, an additional *t*-test did not indicate a significant difference in the rates of correct responses between younger and older adults in Pre trials [*t*_(42)_ = 1.43; *p* < 0.05].

In order to further analyze the effect of Sequence on performance, the percentage decrease in correct responses in Post1, Post2, and Post3 trials (relative to Pre trials) was computed and these normalized data were submitted to a Sequence by Age ANOVA. There was a main effect of Sequence [*F*_(2, 84)_ = 85.1; *p* < 0.001; $\eta_{p}^{2}=0.76$], resulting from the recovery of performance in post-change trials (Figure 2B, right panel). *Post-hoc t*-tests revealed significant differences between all three sequences (Post1: −15.4%; Post2: −8.2%; Post3: −2.7%; all *p* < 0.001). Furthermore, there was a main effect of Age [*F*_(1, 42)_ = 4.6; *p* < 0.05; $\eta_{p}^{2}=0.10$], indicating that the older participants showed a stronger decline in performance in trials following a change than the younger ones (−10.2 % vs. -7.3 %). Even though the ANOVA did not indicate an interaction (*p* > 0.05), additional *t*-tests indicated significant differences between younger and older participants in the percentage decrease of correct responses in Post2 trials [*t*_(42)_ = 2.54; *p* < 0.05] and in Post3 trials [*t*_(42)_ = 2.804; *p* < 0.01], but not in Post1 trials [*t*_(42)_ = 0.97; *p* > 0.05].

### Event-related potentials

**Figure 3:**
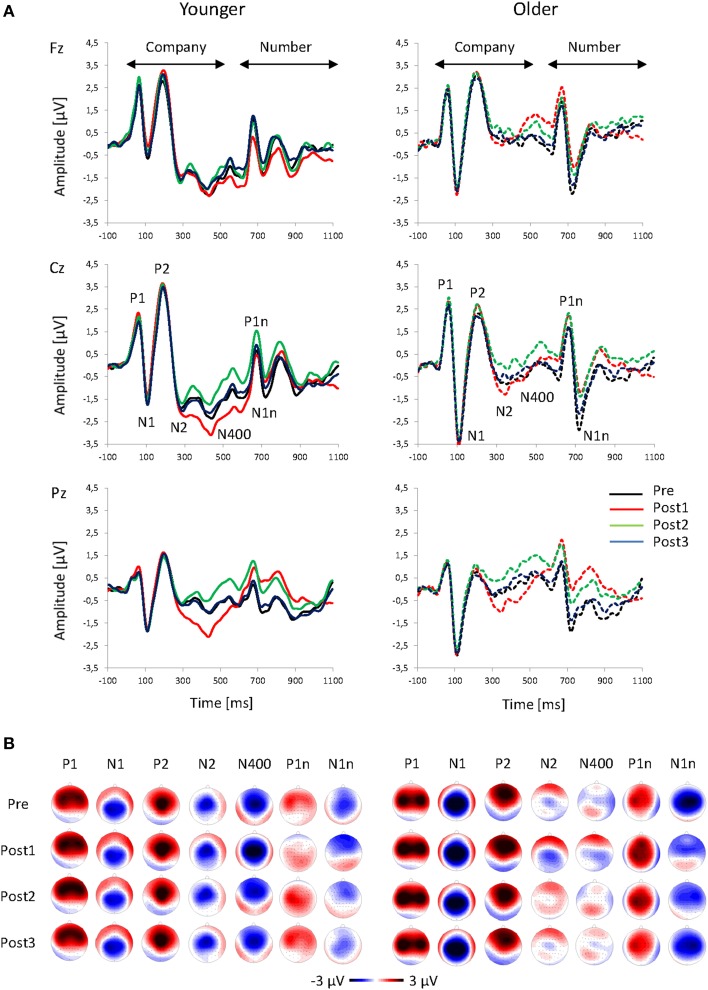
**Grand-average ERPs. (A)** ERPs at Fz, Cz, and Pz plotted as a function of time relative to the speech onset and **(B)** topographies of the ERP components to the onset of the company name (P1, N1, P2, N2, and N400) and value (P1n and N1n) for Pre, Post1, Post2, and Post3 sequences, and for younger and older participants.

The onset of the company names produced a typical fronto-central P1-N1-P2 complex that peaked at 60, 112, and 198 ms, respectively (averaged across all sequences and both age groups; Figure 3). Thereafter, there were two negative peaks at 300 ms (N2) and 429 ms (N400), which were especially pronounced in the younger group. About 600 ms after the company onset, the number onset elicited a second complex of ERPs that mainly consisted of the P1n and N1n components, peaking at 71 and 130 ms after the number onset. In the analyses described below, all these ERP components are compared between groups and sequences.

#### P1

**Figure 4:**
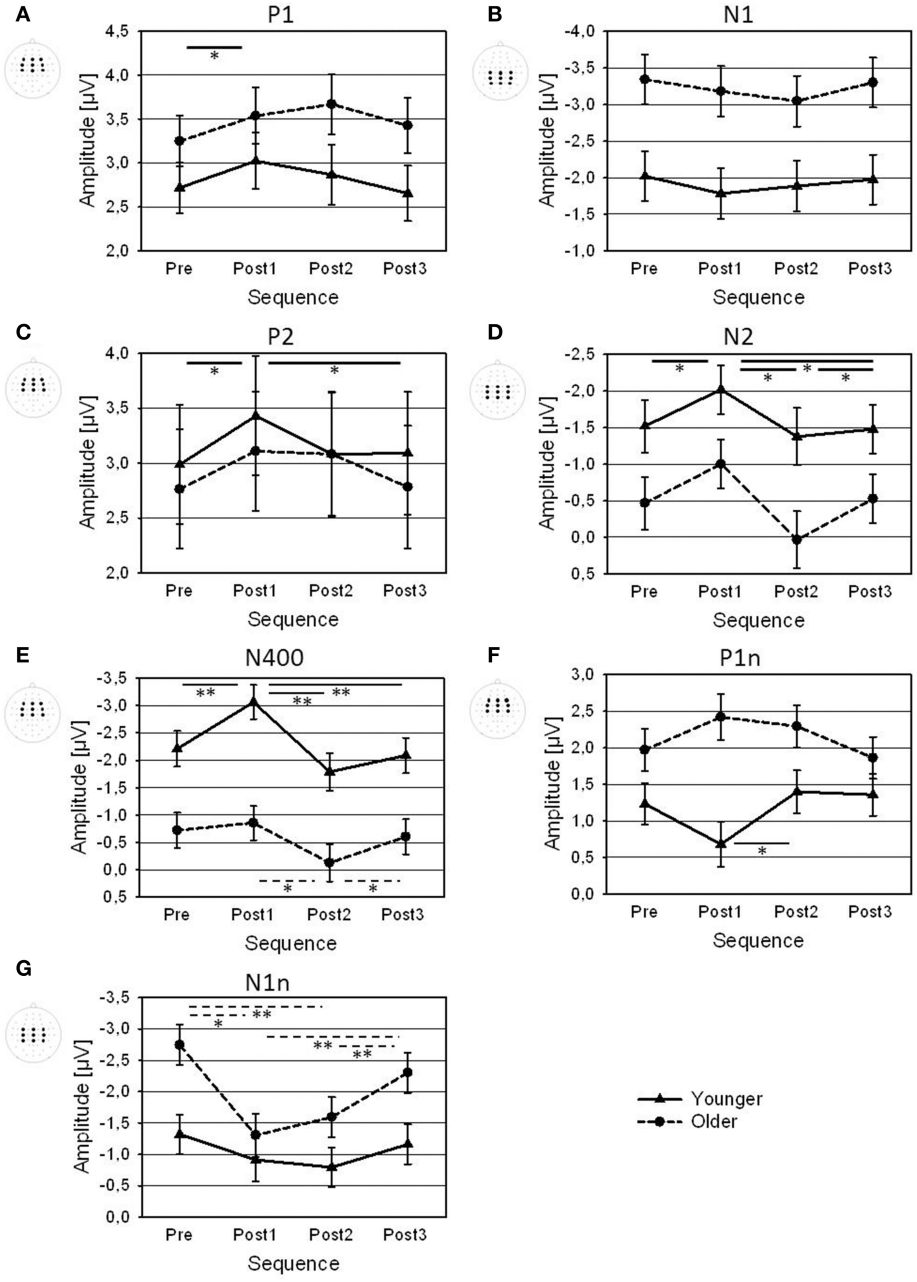
**Amplitudes of (A) P1, (B) N1, (C) P2, (D) N2, (E) N400, (F) P1n, and (G) N1n (averaged across the displayed electrode arrays) for Pre, Post1, Post2, and Post3 sequences, shown for younger and older participants**. Error bars are standard errors across participants (*N* = 22). Significance bars and asterisks indicate significant differences between sequences in the younger group (solid), in the older group (dotted), or averaged across both groups (bold; ^*^p < 0.05; ^**^p < 0.01).

The topography of the P1 showed a frontal positivity for the younger group, and a fronto-central positivity for the older group (Figure 3; Table 1). There was an increase in P1 amplitude immediately after the change that was followed by a decrease (Figure 4A). *Post-hoc t*-tests indicated a larger Post1 than Pre amplitude (*p* < 0.05). There were no effects of Age or Sequence on P1 latency.

**Table 1:** **Main effects (***F***-values and $\eta_{p}^{2}$) of Age and Sequence as well as Age × Sequence interactions on ERP amplitudes (additional factors Frontality and Laterality) and latencies**.

		**P1**		**N1**		**P2**		**N2**		**N400**		**P1n**		**N1n**	
	**df**	***F***	**$\eta_{p}^{2}$**	***F***	**$\eta_{p}^{2}$**	***F***	**$\eta_{p}^{2}$**	***F***	**$\eta_{p}^{2}$**	***F***	**$\eta_{p}^{2}$**	***F***	**$\eta_{p}^{2}$**	***F***	**$\eta_{p}^{2}$**
**AMPLITUDE**															
Age	1, 42	2.3	0.05	7.6^**^	**0.15**	0.1	0.01	8.8^**^	**0.17**	21.7^***^	**0.34**	7.0^*^	**0.14**	4.9^*^	**0.10**
Sequence	3, 126	4.6^**^	**0.10**	2.7^*^	**0.06**	6.4^***^	**0.13**	7.1^***^	**0.15**	3.2^*^	**0.07**	1.4	0.03	19.3^***^	0.32
Age × Sequence	3, 126	1.2	0.03	0.6	0.01	1.1	0.03	1.5	0.03	3.1^*^	**0.07**	5.7^**^	**0.12**	4.9^**^	0.11
Laterality	2, 84	2.4	0.05	21.4^***^	**0.34**	52.2^***^	**0.55**	35.9^***^	**0.46**	10.6^***^	**0.20**	6.3^**^	**0.13**	6.3^**^	**0.13**
Age × Laterality	2, 84	0.3	0.01	0.4	0.01	6.3^**^	**0.13**	1.4	0.03	3.4^*^	**0.08**	1.9	0.04	0.1	0.1
Frontality	2, 84	5.0^**^	**0.11**	8.8^**^	**0.17**	17.5^***^	**0.29**	3.6^*^	**0.08**	4.9^*^	**0.10**	0.4	0.01	8.7^**^	**0.17**
Age × Frontality	2, 84	12.7^***^	**0.23**	4.4^*^	**0.09**	4.7^*^	**0.10**	9.3^**^	**0.18**	0.8	0.02	0.3	0.01	0.3	0.01
Laterality × Frontality	4, 168	6.6^***^	**0.14**	2.9	0.06	14.9^**^	**0.09**	6.1^***^	**0.13**	1.1	0.03	6.2^***^	**0.13**	8.2^***^	**0.16**
Sequence × Laterality	6, 252	1.3	0.03	0.4	0.01	1.4	0.03	2.7^*^	**0.06**	3.1^**^	**0.07**	5.2^***^	**0.11**	3.6^**^	**0.08**
Sequence × Frontality	6, 252	0.6	0.01	1.1	0.02	1.7	0.04	7.7^***^	**0.15**	25.9^***^	**0.38**	7.7^***^	**0.16**	18.7^***^	**0.31**
Age × Lat × Front	4, 168	1.1	0.03	1.4	0.03	4.2^**^	**0.09**	5.3^***^	**0.11**	4.0^**^	**0.09**	4.2^**^	**0.09**	4.6^**^	**0.10**
Age × Seq × Lat	6, 252	1.0	0.02	1.7	0.04	0.9	0.02	2.3^*^	**0.05**	1.6	0.04	0.6	0.01	0.8	0.02
Age × Seq × Front	6, 252	0.7	0.02	0.5	0.01	0.8	0.02	0.3	0.01	0.3	0.01	3.3^**^	**0.07**	1.8	0.04
Seq × Lat × Front	12, 504	1.0	0.02	1.6	0.04	1.6	0.04	0.5	0.01	1.9	0.04	2.1^*^	**0.05**	2.3^**^	**0.05**
Age × Seq × Front × Lat	12, 504	1.9	0.04	2.3^**^	**0.05**	3.0^**^	**0.07**	1.6	0.04	1.4	0.03	0.4	0.01	1.0	0.02
**LATENCY**															
Age	1, 42	0.64	0.02	0.1	0.01	7.7^**^	**0.16**	6.3^*^	**0.13**	0.3	0.01	18.8^***^	**0.31**	0.1	0.1
Sequence	3, 126	0.38	0.01	0.4	0.01	0.1	0.01	2.2	0.06	2.8^*^	**0.06**	0.8	0.2	2.1	0.05
Age x Sequence	3, 126	0.79	0.02	1.5	0.04	0.6	0.01	0.9	0.02	1.2	0.03	1.0	0.02	0.4	0.01

*Significant effects are in bold*.

* p < 0.05;

** p < 0.01;

*** *p < 0.001*.

#### N1

The topography of the N1, showed a centro-parietal negativity and the older group a central negativity (Figure 3; Table 1). Older participants had a much larger N1 than the younger ones. There was a slight decrease in N1 amplitude after a change that appeared to be more pronounced in Post1 trials in the younger group, and in Post2 trials in the older group (Figure 4B). However, separate *Post-hoc t*-tests for the two age groups did not indicate significant differences between sequences, neither for the younger, nor for the older, group. There were no effects of Age or Sequence on N1 latency.

#### P2

The P2 topography showed a central positivity for the younger group, and a fronto-central positivity for the older group (Figure 3; Table 1). Older participants showed a broader distribution than the younger ones. There was an increase in P2 amplitude after the change followed by a decrease. *Post-hoc t*-tests indicated a larger P2 amplitude in Post1 trials than in Pre trials and in Post3 trials (both *p* < 0.05). This pattern differed between age groups: While the increase in Post1 amplitude was pronounced over mid-frontal scalp areas mainly in the younger group, in the older group the increase in P2 amplitude was visible in Post1 and Post2 trials and showed a broader topographical distribution (Figure 3B). In addition, P2 latency was generally shorter in younger, than older, subjects (192 vs. 206 ms).

#### N2

The topography of the N2 showed a mid-central negativity (Figure 3; Table 1) that was much stronger in the younger, than in the older, group, in particular over frontal scalp areas). The N2 amplitude increased in Post1 trials and decreased again in Post2 and Post3 trials (Figure 4D). In both age groups, this pattern was especially pronounced over centro-parietal areas, where *Post-hoc t*-tests indicated a significant N2 increase in Post1 trials (relative to Pre, Post2, and Post3 trials), and a N2 decrease in Post 2 trials (relative to Post3 trials; all *p* < 0.05). Younger subjects had a shorter N2 latency than the older ones (293 vs. 307 ms).

#### N400

The N400 topography showed a mid-fronto-central negativity (Figure 3; Table 1), which was stronger in the younger, than in the older, group. There was an increase in N400 amplitude after a change that was followed by a decrease (Figure 4E). This pattern was more pronounced in younger, than older, subjects. Accordingly, *Post-hoc t*-test indicated a significant increase in N400 amplitude over central areas in Post1 trials (relative to Pre, Post2, and Post3 trials) in the younger group (all *p* < 0.005), while amplitudes in Post2 trials were reduced relative to Post1 and Post3 trials in the older group (both *p* < 0.05). There was a slight effect of Sequence on N400 latency, although *Post-hoc t*-tests did not indicate significant differences between sequences. Also, there was no effect of Age on N400 latency.

#### P1n

The P1n to the onset of number had a maximum over left-central areas (Figure 3; Table 1), and was stronger in older, than younger, subjects. Moreover, the age groups differed in the effect of Sequence (Figure 4F): While younger subjects showed a P1n decrease in Post1 trials (and an increase thereafter), older subjects showed a slight P1n increase in Post1 and Post2 trials. *Post-hoc t*-tests indicated significant differences in P1n amplitude between Post1 and Post2 trials in the younger group (*p* < 0.05), but no differences in the older group. Older subjects had an earlier P1 than younger ones (664 vs. 677 ms).

#### N1n

The N1n topography showed a fronto-central negativity (Figure 3; Table 1). Older subjects had a larger N1n than the younger ones, and the N1n showed a broader distribution across the scalp. There was a strong decrease in N1n amplitude after the change, with a following recovery. This pattern was more pronounced in the older, than younger, group. Accordingly, *Post-hoc t*-tests indicated an N1n reduction in Post1 and Post2 trials relative to Pre and Post3 trials in the older group (all *p* < 0.005), but no differences in the younger group. There were no effects of Age or Sequence on N1n latency.

### Difference waves

**Figure 5:**
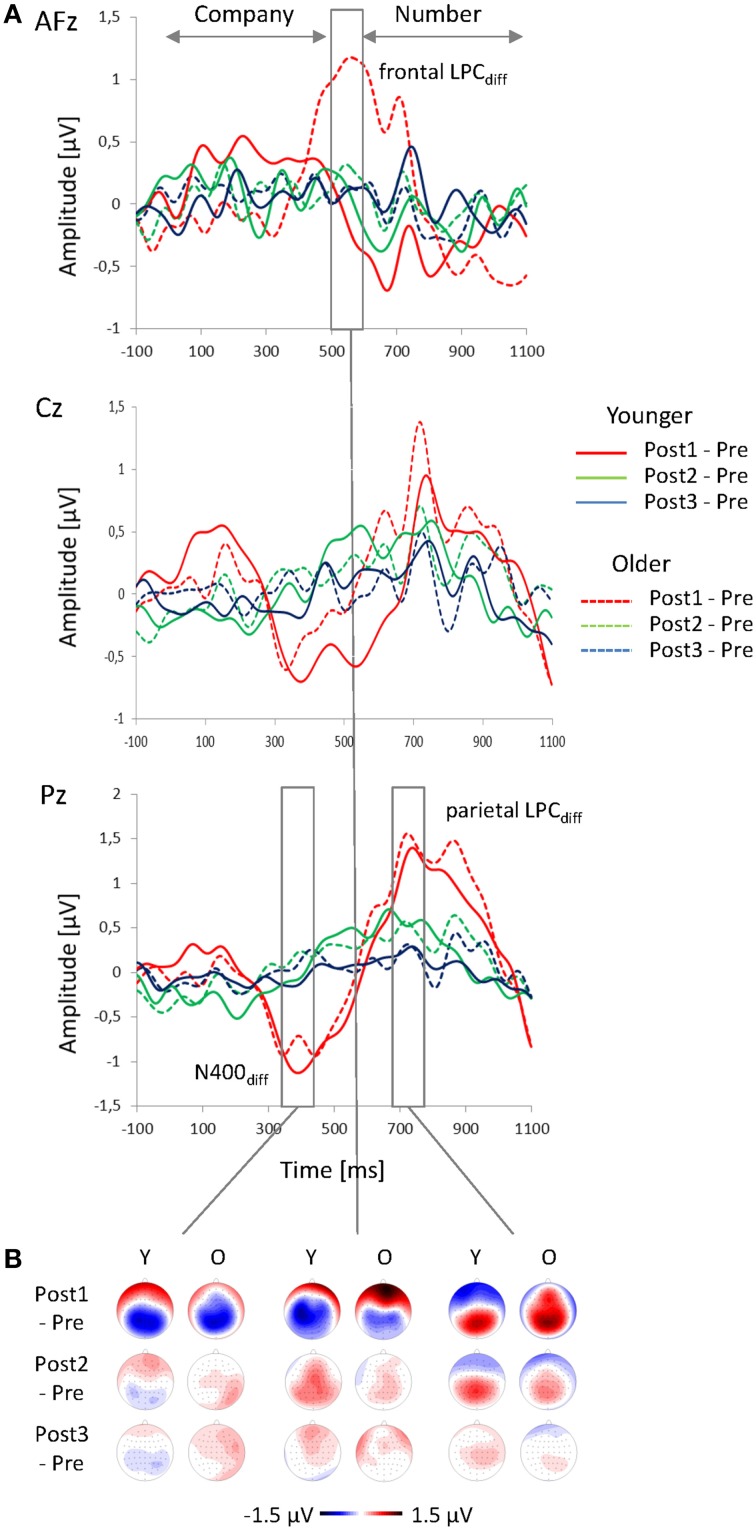
**Change-related ERPs. (A)** Difference waveforms (Post minus Pre) at AFz, Cz, and Pz plotted as a function of time relative to the speech onset and **(B)** topographies of the change-related ERPs (N400_diff_, frontal and parietal LPC_diff_) for Post1, Post2, and Post3 sequences, shown for younger and older participants.

The analysis of the difference waveforms (Post minus Pre) mainly revealed two deflections, that were most pronounced over parietal scalp areas: a negative deflection peaking at about 390 ms after speech onset (N400_diff_) and a late positive component (parietal LPC_diff_) peaking at about 740 ms (Figure 5). In addition, the older subjects showed a prominent positive deflection over right-frontal scalp regions (frontal LPC_diff_) that peaked about 550 ms after speech onset and that was not found in the younger group. For analysis of these deflections, the mean amplitudes within 100-ms time windows around the peak latencies were computed and subjected to Four-Way ANOVAs with the between-subject factor Age (younger, older) and the within-subject factors Sequence (Post1, Post2, Post3), Frontality, and Laterality.

#### N400_diff_

**Figure 6:**
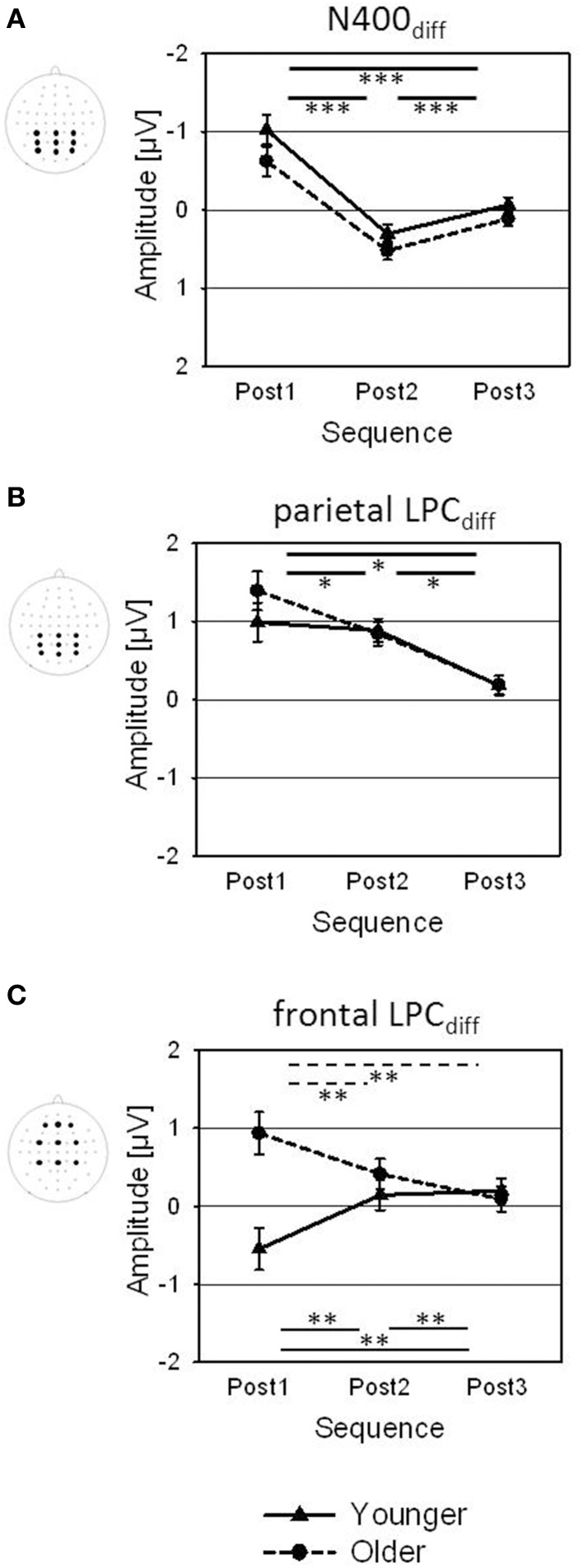
**Amplitudes of the change-related ERPs [(A) N400_diff_, (B) parietal and (C) frontal LPC_diff_; averaged across the displayed electrode arrays] for Post1, Post2, and Post3 sequences, shown for younger and older participants**. Error bars are standard errors across participants (*N* = 22). Significance bars and asterisks indicate significant differences between sequences in the younger group (solid), in the older group (dotted), or averaged across both groups (bold; ^*^p < 0.05; ^**^p < 0.01; ^***^p < 0.001).

The topography of the N400_diff_ showed a parietal negativity for the younger subjects, and a centro-parietal negativity for the older adults (Figure 5B; Table 2). The N400_diff_ mainly occurred in Post1 trials (mean −0.82μV, SE 0.14 μV), turned into a slight positivity in Post2 trials (0.41 μV, SE 0.09 μV) and disappeared in Post3 trials (0.02 μV, SE 0.07 μV; Table 1; Figure 6A). *Post-hoc t*-test indicated significant differences between all three sequences (all *p* < 0.001). The N400_diff_ in Post1 trials was lateralized to the left in the younger group and to the right in the older group. No further main effect or interaction was found.

**Table 2:** **Main effects (***F***-values and η${}_{p}^{2}$) of Age and Sequence as well as Age × Sequence interactions on amplitudes (additional factors Frontality and Laterality) of the difference ERP (Post minus Pre)**.

**Amplitude**		**N400**_**diff**_		**Parietal LPC**_**diff**_		**Frontal LPC**_**diff**_	
	**df**	***F***	***$\eta_{p}^{2}$***	***F***	***$\eta_{p}^{2}$***	***F***	**η${}_{p}^{2}$**
Age	1, 42	3.2	0.07	0.3	0.01	3.9	0.09
Sequence	2, 84	58.3^***^	**0.58**	32.6^***^	**0.44**	4.0^*^	**0.09**
Age × Sequence	2, 84	0.5	0.01	1.8	0.04	12.7^***^	**0.23**
Laterality	2, 84	3.0	0.7	5.7^**^	**0.12**	14.5^***^	**0.26**
Age × Laterality	2, 84	0.1	0.1	0.3	0.01	0.2	0.01
Frontality	2, 84	2.3	0.5	3.9^*^	**0.09**	0.4	0.01
Age × Frontality	2, 84	5.1^**^	**0.11**	3.9^*^	**0.09**	3.0	0.07
Laterality × Frontality	4, 168	1.5	0.04	0.7	0.02	3.1^*^	**0.07**
Sequence × Laterality	4, 168	0.8	0.02	1.4	0.03	2.7^*^	**0.06**
Sequence × Frontality	4, 168	1.6	0.04	8.3^***^	**0.16**	29.8^***^	**0.42**
Age × Lat × Front	4, 168	1.4	0.03	1.5	0.04	1.1	0.03
Age × Seq × Lat	4, 168	3.7^*^	**0.08**	1.7	0.04	0.3	0.01
Age × Seq × Front	4, 168	0.3	0.01	2.7^*^	**0.06**	2.9^*^	**0.07**
Seq × Lat × Front	8, 336	1.3	0.03	1.2	0.03	2.1^*^	**0.05**
Age × Seq × Front × Lat	8, 336	1.6	0.04	1.8	0.04	2.0	0.05

*Significant effects are in bold*.

* p < 0.05;

** p < 0.01;

*** *p < 0.001*.

#### Parietal LPC_diff_

The topography LPC_diff_ showed a mid-parietal maximum for the younger group, and a mid-centro-parietal maximum for the older group (Figure 5B; Table 2). The maximum amplitude was measured in Post1 trials (mean 1.19 μV, SE 0.18 μV) and decreased in Post2 trials (0.86 μV; SE 0.11 μV) and in Post3 trials (0.18 μV, SE 0.09 μV). *Post-hoc t*-tests indicated significant differences between all three sequences (all *p* < 0.05). No further main effect or interaction was found.

#### Frontal LPC_diff_

In the older group, a strong frontal LPC_diff_ with a right anterior-frontal maximum occurred in the time window between 500 to 600 ms after speech onset, i.e., immediately before the number stimulus started. This frontal LPC_diff_ did not occur in the younger group, where a left-lateralized centro-parietal negativity was found (Figure 5B; Table 2). The positivity and the negativity were apparent only in Post1 trials and disappeared in Post2 and Post 3 trials. Moreover, there were Age × Sequence (Figure 6C) and Age × Sequence × Frontality interactions. Separate *Post-hoc t*-tests were conducted for the older group at anterior-frontal electrodes and for the younger group at centro-parietal electrodes. These *t*-tests indicated a significantly larger frontal LPC_diff_ in Post1 trials (mean 1.25 μV, SE 0.26 μV) than in Post2 trials (0.27 μV, SE 0.19 μV) and in Post3 trials (0.09 μV, SE 0.18 μV) for the older group (both *p* < 0.005), but no difference between Post2 and Post3 trials (*p* > 0.05). For the younger group, the amplitudes in Post1 (−0.62 μV, SE 0.29 μV), Post2 (0.83 μV, SE 0.18 μV), and Post3 trials (0.14 μV, SE 0.15 μV) all differed significantly (all *p* < 0.01). Finally, to test whether the anterior-frontal positivity of the older group was related to performance in speech perception, the Pearson correlation between the amplitude of the frontal LPC_diff_ (averaged across the anterior-frontal electrodes) and the individual rates of correct responses (averaged across Post1, Post2, and Post3 trials) was computed. There was a slight, but significant positive correlation (*r* = 0.43; *p* < 0.05), indicating that a higher rate of correct responses was associated with a greater frontal LPC_diff_.

### Summary of ERP results

Changes in speaker settings resulted in increased P1, P2, N2, and N400 amplitudes. Moreover, the difference waveforms (Post minus Pre) revealed a pronounced N400_diff_ and a parietal LPC_diff_ complex in trials immediately after the change, which decreased in amplitude between trials Post1 and Post3. Older participants showed larger N1, P1n, and N1n amplitudes than younger participants, whereas younger participants showed larger N2 and N400 amplitudes than the older participants. In addition, the younger group showed an increase in N400 amplitude after the change that was not found in the older group. The older group showed a decrease in N1n amplitude after the change and a prominent frontal LPC_diff_ that did not occur in the younger group.

## Discussion

A change in speaker settings in a multi-speaker scenario declined the performance in speech perception immediately after the change, as was evident by the profound decrease in the rate of correct responses. This decline is in accordance with the switch costs assumed by Koch et al. (2011). The present findings are also in line with results of a recent study in which switch costs associated with conversational turn-taking were investigated in a sentence-recall task in younger adults (Lin and Carlile, 2015). Here, relative to a non-changing condition, shifts in spatial attention from one speaker position to another within a sentence decreased the performance of word recall by about 11%. The switch costs could result from re-selection of the target speaker among the concurrent speech information and from re-focusing of attention on the new target. As suggested by Shinn-Cunningham and Best (2008), it may took some time for the processes of re-selection and re-focusing to be performed, as indicated by a rather slow recovery of performance in trials following a change. In fact, the rate of correct responses was reduced even in Post2 trials and re-approached the pre-change level not until Post3 trials. These results also show that continuity in an auditory scene increases the efficacy of object selection and focusing of attention (Best et al., 2008), at least until the next change occurred.

Similar as in our recent study (Getzmann et al., 2015a), the older participants showed a slightly worse performance than the younger ones in general, suggesting that they had more difficulties extracting the relevant information out of the stream of concurrent speech stimuli. However, this difference failed to reach statistical significance. Most important is that the older adults had more difficulties with a change in speaker setting, as indicated by the stronger decline in performance in trials following a change. While the age groups did not significantly differ before and immediately after a change, the older group showed a stronger decrease in performance (relative to the pre-change level) than the younger group in Post2 and Post3 trials. In fact, even in the third sequences after a change the older group performed worse than the younger group, indicating that it obviously took longer for the older participants to recover from the distraction resulting from the change.

This observation is in line with previous findings, in which the processing of relevant auditory stimuli before and after the occurrence of a task-irrelevant event was investigated in younger and older adults. In a cross-modal oddball task, the increase in response times to standard stimuli following a distracting deviant stimulus (relative to standards preceding the deviant) was larger in older, than in younger, participants (Parmentier and Andrés, 2010). Recent ERP findings from a dynamic speech perception task indicated that this prolonged post-deviance distraction was associated with a reduced and delayed attentional and inhibitory control, affecting both the handling of a distracting event as such and the auditory input following the distracting event (Getzmann et al., 2014b). The results also suggested that the processing of a task-irrelevant, distracting stimulus feature (i.e., a change in the spatial location of a target speaker) was prolonged in the older group (Getzmann and Wascher, under review). The analysis of the ERPs in the present study revealed that age-related differences in the handling of changes in speaker settings were associated with differences in electrophysiological measures. These differences were mainly found in the later components (N400, P1n, N1n) and frontal LPC_diff_, while modulation of the earlier components (P1, N1, P2, and N2) and the parietal N400_diff_ and LPC_diff_ by changes in speaker settings were quite similar in younger and older subjects (cf. Figures 4, 6).

### Age-related differences in change processing in N400, P1n, and N1n

The younger group had a greater fronto-central N400 amplitude and, even more important, showed an increase in N400 amplitude after a change in speaker settings that was not found in the older group. Age-related declines in N400 have also been found in previous studies, e.g., in semantic categorization tasks (Woodward et al., 1993; Kutas and Iragui, 1998; Federmeier et al., 2002), in a multi-speaker word-pair semantic categorization task (Davis et al., 2013), and in a speech-in-noise perception task, in which the N400 was nearly absent in the older group and in which the N400 mainly occurred in a multi-speaker condition (Getzmann et al., 2015a). The N400 is assumed to be a correlate of processing of meaningful (or potentially meaningful) stimuli that is typically linked to language perception. It has been related to a wide range of cognitive functions, comprising orthographic and phonological analysis such as word recognition, integration of a word's meaning into the preceding context, as well as activation of access to semantic memory within a comprehension network (for review, see Kutas and Federmeier, 2011). The N400 has also been related to inhibition (Debruille, 2007). In a study of knowledge inhibition, the N400 elicited by distractors was greater when distractors had to be ignored than attended. Moreover, participants who performed well in ignoring the distractors had a larger N400 amplitude than poor ignorers (Debruille et al., 2008). In a related study, words elicited a more negative N400 when their meanings were task inappropriate than when these meanings had to be used (Shang and Debruille, 2013). These results were interpreted in a way that the N400 could reflect processes of inhibition of representations that have been inappropriately activated. In the present task, the increase in N400 amplitude that was found after a change in the younger group could indicate increased speech processing and enhanced extraction and processing of meaningful speech information as well as increased inhibition of the concurrent speech content in trials following a change. The absence of an N400 increase in the older group, on the other hand, suggests that these subjects did not adapt these processes to the changing speaker settings.

The P1n and N1n components were elicited by the second speech stimulus, i.e., the numerals following the company names. Older participants had an overall greater P1n than the younger ones. Moreover, younger subjects showed a P1n decrease in Post1 trials (and an increase thereafter), which was not found in the older group. A decrease in P1 amplitude has been observed when a target stimulus was presented at a location contralateral to the side of attention (e.g., Mangun and Hillyard, 1991; Van Voorhis and Hillyard, 1977). Within the theoretical framework of the “cost of attention” (Luck et al., 1994), the P1 decrease could indicate the cost of attending to an incorrect location. Thus, the suppression of P1n could be interpreted as representing the process of stopping to attend to one location or speaker and of shifting the attention to the location or speaker where the target stimulus was currently located. This interpretation should be treated with caution, however, because the decrease of the Post1 P1 could—at least in part—result from a temporal overlap with the increase in Post1 N400 that was observed in the younger, but not in the older, group (cf. Figure 3).

Older participants showed a strong decrease in N1n amplitude after a change and a recovery thereafter, while no significant decrease in N1n was found in the younger group. Assuming the greater N1n of the older group (as seen in the N1 and in the pre-change N1n; cf. Figures 4B,G) to reflect attentional processes related to a compensatory increase of early stimulus processing, the decline in N1n observed after a change could indicate that older adults had difficulties to maintain this activation in the trials following a change.

### Change-specific processes as revealed by difference waveforms

The subtraction of post-change waveforms from the pre-change waveforms eliminated genuine processes of speech perception (as were present in pre- and post-change trials) and allowed to study ERPs related to the processes of changes in speaker setting. There was a pronounced frontal positivity (the frontal LPC_diff_) that only occurred in the older group. The frontal LPC_diff_ had a right anterior-frontal maximum and was most pronounced in Post1 trials and disappeared in Post2 and Post 3 trials. A frontal LPC_diff_ was also observed by Davis and Jerger (2014) who related it to an age-related frontal shift that is typically found in older populations (for review, see Friedman et al., 1997). In the present context, the frontal LPC_diff_ could indicate a stronger orientation to (and perhaps distraction by) the change in speaker setting of the older participants that—consequently—would result in a prolonged capturing of attention toward the task-irrelevant change in speaker settings. However, there was a significant positive correlation of the amplitude of frontal LPC_diff_ and speech perception of older participants, indicating that higher individual performance came along with a greater frontal activation. This is in contrast to the assumption that the frontal LPC_diff_ reflects a negative effect, i.e., in form of a distraction by the change in speaker setting. It appears more plausible that an age-related compensatory mechanism was at work that has also been observed in related speech-perception tasks for the fronto-central P2 (Getzmann et al., 2015b) and P3a components (Getzmann and Falkenstein, 2011). Increased prefrontal activation is usually related to the allocation of attentional resources, and the frontal LPC_diff_ could reflect a more effortful processing of speech stimuli, based on recruitment of frontal areas. Consistent with the decline-compensation hypothesis (Wingfield and Grossman, 2006), the frontal LPC_diff_ can therefore be interpreted within the theoretical framework of a compensation approach (e.g., Cabeza et al., 2002; for review, Schneider et al., 2010), in which deficits in speech perception are compensated by increased of allocation of general processing resources.

In addition to the frontal LPC_diff_, there was a parietal negativity and positivity (i.e., the parietal N400_diff_ and LPC_diff_, respectively) that were observed in both age groups. As already discussed above, the N400 may be related to different aspects of speech perception (for review, see Kutas and Federmeier, 2011). The pronounced N400_diff_ found here could be associated with the mismatch between the current and the expected linguistic input that occurred when the target speaker changed. There is evidence that a repeated presentation of words reduces the N400, whereas a violation of the repetition increases the N400 (for review, see Kutas et al., 2006). The mismatch between the expected target speaker/target location and the current speaker setting could have triggered an increase in speech processing, that is, an enhanced phonological analysis of the linguistic input, possibly associated with the search of the relevant information after the target speaker has changed. Here, differences between the N400_diff_ (as observed after a change) and the N400 (as observed in each trial) should be noted: The N400_diff_ has a parietal topography and occurred in younger and older adults, while the N400 showed a fronto-central topography and was much stronger in the younger group. Thus, the processes reflected by the parietal N400_diff_ did obviously not differ between the younger and older group, while the processes reflected by the N400 appeared to be declined in the older group. However, further research will be necessary to determine the specific characteristics of the change-related N400_diff_ in the context of speech perception in multi-speaker environments.

The N400_diff_ was followed by the parietal LPC_diff_. A similar observation has recently been reported in a multi-speaker word-pair semantic categorization task, in which younger and middle-aged adults responded to an attended stream of words while ignoring competing speech from a different location (Davis and Jerger, 2014). The LPC is usually found to be maximal over parietal scalp areas and is assumed to reflect decision-making and target selection (Picton, 1992; Kok, 1997) as well as context updating and evaluation and processing of stimulus meaning (Juottonen et al., 1996). In the present task, the parietal LPC_diff_ could reflect a shift of attention toward the new target speaker and/or location. In this regard, the role of posterior parietal cortex (PPC) for voluntary attention switching has to be discussed. There is evidence from neuroimaging studies that switching attention in the auditory domain recruits similar cortical networks as engaged by switching attention between visual objects (Corbetta et al., 2008). When listeners switched attention between two streams of different voices or between the two ears, activation in the PPC was higher than in a non-switch condition (Shomstein and Yantis, 2006). Similarly, the right temporo-parietal junction was more engaged in switched, than non-switched, trials (Larson and Lee, 2013, 2014; for review, see Lee et al., 2014). This finding is consistent with evidence from vision, in which the right temporo-parietal junction operates as a “filter” for incoming stimuli by suppressing attention switching to distractors (Shulman et al., 2007) and by acting as a “circuit breaker” to trigger the spatial reorientation of the focus of attention to the new speaker and location of interest (Corbetta and Shulman, 2002). Although it is clear that ERP topographies cannot directly be related to underlying brain structures, it might be conceivable that the parietal N400_diff_ and LPC_diff_ reflect electrophysiological correlates of such processes. Given that there were no differences between younger and older participants, it seems as if these processes were preserved in the older group.

### Effects of age and change processing on P1, N1, P2, and N2

The P1 was increased in trials following a change, relative to pre-change level, independent of the subjects' age. The P1 is known to be elicited relatively early in the auditory cortical processing stream (e.g., Grunwald et al., 2003) and is mainly driven by physical characteristics of the auditory stimulus. Given that changes in speaker settings occurred in each trial (as the concurrent speaker voices and locations changed between trials; cf. Figure 1B), it appears unlikely that changes in the physical characteristics of the target speaker were related to the increase in P1. Rather, the increase in P1 amplitude suggests that the early stimulus processing was affected by a (pre-)attentional detection of a change in the speaker scenario (for evidence of an attention-based modulation of the visual P1, see, e.g., Luck et al., 2000).

While there were no differences in P1 amplitudes, the N1 was greater in older, than younger, participants. The N1 is usually regarded as a correlate of early, automatic processing of incoming auditory stimuli (Näätänen and Picton, 1987) that depends on early attentional processes (Hillyard et al., 1973; Wascher et al., 2009; Wascher and Beste, 2010; Schneider et al., 2012; for review, see Luck et al., 2000; Eimer, 2014). Within a theoretical framework of an early selection model of attention, the N1 could reflect a sensory gating mechanism of attention, which facilitates the further processing of the relevant stimulus. The greater N1 of the older participants is in line with previous studies (e.g., Yordanova et al., 2004; Getzmann et al., 2015a) and could indicate stronger early attentional processes that might be interpreted in accordance with the decline-compensation hypothesis (Wingfield and Grossman, 2006; Schneider et al., 2010). Given that there was no effect of sequence, this appeared to be a more general strategy of the older participants that was not modulated by changes in speaker settings.

In both age groups, the P2 and N2 amplitudes increased in trials following a change and decreased thereafter. The functional significance of the P2 is still not fully understood yet, although this component is usually assumed to reflect processes of stimulus evaluation, indexing some aspects of attentional allocation or stimulus classification (Potts, 2004). The N2 is assumed to reflect control processes in general (for reviews, see Patel and Azzam, 2005; Folstein and Van Petten, 2008) and has been related to conflict processing or inhibitory control of irrelevant information (e.g., Falkenstein et al., 2002; Melara et al., 2002; Bertoli et al., 2005). In the present context, the greater P2 and N2 components could reflect the allocation of attentional resources and the inhibition of the concurrent speech stimuli that was increased when the target speaker changed. This interpretation is in accordance with results of neuroimaging studies in which correlations between the activation of fronto-temporal cortical areas and intelligibility of distorted speech stimuli were found (Davis and Johnsrude, 2003; Hannemann et al., 2007; MacDonald et al., 2008; Obleser and Kotz, 2010).

The P2 did not differ between age groups in amplitude, but had a more frontal topography and was delayed in the older, than in the younger, group. This frontality is in line with the PASA hypothesis (posterior–anterior shift with aging), holding that aging is associated with increasing activity over prefrontal areas that may reflect functional compensation (Davis et al., 2008). In contrast, the younger group showed a greater and earlier N2 than the older group, which is in line with previous studies in which an overall N2 reduction was observed with aging (e.g., Anderer et al., 1996; Wascher et al., 2011; Wascher and Getzmann, 2014). This could indicate a less efficient inhibitory control over the concurrent speech information in the older group, which may be related to an overall inefficiency to suppress neural activity associated with irrelevant and distracting information (for review, see Gazzaley and D'Esposito, 2007), according to the inhibitory deficit hypothesis (Hasher and Zacks, 1988). In line with this interpretation, older adults showed—in contrast to the younger group—a pronounced frontal LPC in the difference waveform, possibly reflecting increased attention to, or distraction by, a change in speaker location (as discussed in Change-specific Processes as Revealed by Difference Waveform). It should be noted, however, that the reduction of N2 in the older group was not modulated by sequence, suggesting that the inhibitory deficits were not directly related to the deficits found in speech perception after a change.

### Conclusions

Changes in speaker settings in a complex multi-speaker environment resulted in a decline of the performance in speech perception that was more serious in older, than younger, adults. This decline is based on different characteristics of the aging brain: While the change-related processes of increasing effort in speech processing and attention switching (as reflected by the parietal N400_diff_ and LPC_diff_) appeared to remain unaffected by age, older adults showed a stronger allocation of mental resources to the processing of the speech stimuli after a change in the auditory environment (indicated by the frontal LPC_diff_) and obviously had a lesser flexible adaptation of speech processing and inhibition of concurrent speech content (indicated by the N400) than younger adults. In addition, older persons showed difficulties in maintaining compensatory activation (indicated by the N1) that might subserve speech perception in non-switch trials.

### Conflict of interest statement

The authors declare that the research was conducted in the absence of any commercial or financial relationships that could be construed as a potential conflict of interest.
